# Survival in patients with stage IV noncardia gastric cancer - the influence of DNA ploidy and *Helicobacter Pylori* infection

**DOI:** 10.1186/1471-2407-12-264

**Published:** 2012-06-21

**Authors:** John Syrios, Stavros Sougioultzis, Ioannis D Xynos, Nikolaos Kavantzas, Christos Kosmas, George Agrogiannis, John Griniatsos, Ioannis Karavokyros, Emmanouil Pikoulis, Efstratios S Patsouris, Nikolas Tsavaris

**Affiliations:** 1 Department of Pathophysiology, Oncology Unit, Laikon General Hospital, Athens University School of Medicine, Athens, Greece; 2 1st Department of Pathology, Laikon General Hospital, Athens University School of Medicine, Athens, Greece; 3 2nd Department of Medical Oncology, Metaxa Cancer Hospital, Piraeus, Greece; 4 1st Department of Surgery, Laikon General Hospital, Athens University School of Medicine, Athens, Greece

## Abstract

**Background:**

Palliative surgery followed by postoperative chemotherapy is a challenging approach in the treatment of stage IV gastric cancer yet patients must be carefully selected on the basis of likely clinical benefit.

**Methods:**

The records of 218 patients with histological diagnosis of gastric adenocarcinoma who underwent palliative surgery followed by postoperative chemotherapy were retrospectively reviewed. Twelve potential prognostic variables including tumour DNA index and serum IgG anti- *Helicobacter pylori* (HP) antibodies were evaluated for their influence on overall survival by multivariate analysis.

**Results:**

The median survival was 13.25 months [95% Confidence Interval (CI) 12.00, 14.50]. Three factors were found to have an independent effect on survival: performance status (PS) [PS 60–70 *vs*. 90–100 Hazard Ratio (HR) 1.676; CI 1.171-2.398, p = 0.005], liver metastases (HR 1.745; CI 1.318-2.310, p < 0.001), and DNA Index as assessed by Image cytometry (2.2-3.6 *vs.* >3.6 HR 3.059; CI 2.185-4.283, p < 0.001 and <2.2 *vs.* >3.6 HR; 4.207 CI 2.751-6.433 <0.001). HP infection had no statistically significant effect on survival by either univariate or multivariate analysis.

**Conclusion:**

Poor pre-treatment PS, the presence of liver metastasis and high DNA Index were identified factors associated with adverse survival outcome in patients with Stage IV gastric cancer treated with palliative gastrectomy and postoperative chemotherapy. HP infection had no influence on survival of these patients.

## Background

Gastric adenocarcinoma is an aggressive tumour accounting for the second leading cause of cancer specific mortality worldwide. Surgical resection remains the main curative treatment for gastric cancer although it remains applicable in only 10–20% of cases who present with limited stage disease [1].

The role of palliative gastrectomy in stage IV gastric cancer [defined as M1 and any T or N according to the American Joint Commission of Cancer (AJCC, 7^th^ edition) criteria] is still controversial. A randomized controlled trial has started in both Japan and Korea aiming to evaluate the role of gastrectomy in the management of advanced gastric cancer and results are awaited [2]; nevertheless, a number of studies, including one from our group, have shown a survival benefit [3-6].

Furthermore, systemic chemotherapy for advanced gastric adenocarcinoma has proven of limited value due to the low response rates and severe adverse effects [4-8]. However, as both palliative surgery and postoperative chemotherapy have evolved as independent prognostic factors for survival previously [6-8], it would be important to identify factors which could predict survival benefit in patients selected for a combined treatment with palliative gastrectomy followed by systemic chemotherapy.

In this study we explored the above notion by performing an analysis of prognostic factors in a subgroup of patients from our previously described cohort who received palliative surgery followed by postoperative chemotherapy. The pool of prognostic factors investigated was expanded with the addition of tumour DNA content (DNA Index) and *H. Pylori* (HP) infection.

## Methods

### Patients and data sources

The patient cohort has been described in detail elsewhere [6]. Briefly, this included 311 consecutive patients with a histological diagnosis of gastric adenocarcinoma (noncardia) from a single Oncology Center, treated outside of clinical trials. In this subgroup analysis data from 218/311 patients who underwent palliative surgery followed by chemotherapy [Leucovorin modulated 5-Fluorouracil (5-FU), or combination chemotherapy regimens including combination treatments based on Epirubicin, Oxaliplatin and Capecitabine according to evolving protocols] were retrospectively reviewed for prognostic factors affecting overall survival (OS). OS was calculated from time of diagnosis to death due to gastric cancer-related complications. Records with complete data (for the parameters used as prognostic factors) were included in the analysis. The study was approved by the Ethical Committee for Research Projects of Laiko Hospital, Athens, Greece.

### Prognostic variables

Twelve putative clinicopathological prognostic variables were selected for this analysis (Table 1). Patient-related factors included age (≤60 years or >60 years), gender, and pre-treatment performance status (PS) according to the Karnofsky Performance Status Scale Index. Tumor- related factors included histological grading according the World Health Organisation (WHO) system, location of metastases: local invasion, lymph nodes, liver, lung, ovaries, bone, abdomen/peritoneum; and biochemical/serological parameters. For the latter, group categorizations were used: for carcinoembryonic antigen (CEA): normal ≤ 5 ng/dl *vs.* elevated >5 ng/dL; for cancer antigen 19-9 (CA 19-9): values ≤ 30 U *vs.* >30 U; for cancer antigen 72–4 (CA 72–4): normal ≤ 7 U/ml *vs*. elevated >7U/ml; for C-reactive protein (CRP): normal ≤5 mg/dl *vs.* elevated >5 mg/dl; for Albumin normal >3.4 g/dL *vs.* decreased ≤3.4 g/dL and for HP infection infected vs. not infected; for DNA Index, group categorization was also applied for analytical purposes: <2.2 (Low), 2.2-3.6 (Intermediate), >3.6 (High).

**Table 1:** Patient Characteristics

**Descriptive Statistics**		**Count**	**%**
Age	≤60	101	46.3%
	>60	117	53.7%
Gender	Male	147	67.4%
	Female	71	32.6%
	60-70	66	30.3%
Karnofsky PS	80	84	38.5%
	90-100	68	31.2%
	1	11	5.0%
Histological Grade	2	129	59.2%
	3	78	35.8%
Lymph Node Metastasis	No	45	20.6%
	Yes	173	79.4%
Liver Metastasis	No	112	51.4%
	Yes	106	48.6%
Lung Metastasis	No	201	92.2%
	Yes	17	7.8%
Ovary Metastasis	No	197	90.4%
	Yes	21	9.6%
Bone Metastasis	No	211	96.8%
	Yes	7	3.2%
Peritoneal dissemination	No	122	56.0%
	Yes	96	44.0%
Albumin (normal range >3.4 g/dl)	Normal	137	62.8%
	Low	81	37.2%
CRP (normal range ≤5 mg/dl)	Normal	132	60.6%
	High	86	39.4%
HP	No	142	65.1%
	Yes	76	34.9%
CEA	≤5	112	51.4%
	>5	106	48.6%
CA19-9	≤30	86	39.4%
	>30	132	60.6%
CA 72-4	≤7	40	18.3%
	>7	178	81.7%
	<2.2	88	40.4%
DNA Index	2.2-3.6	88	40.4%
	>3.6	42	19.2%

*PS* Performance Status, *CRP* C-Reactive Protein, *HP Helicobacter Pylori*, *CEA* Carcinoembryonic Antigen *CA 19–9* Cancer Antigen 19–9, *CA 72–4* Cancer Antigen 72–4.

### DNA image cytometry (DNA Index)

For DNA measurements the Feulgen staining technique was applied which labels DNA as magenta and the intensity of the stain is directly proportional to the amount of DNA present. Briefly, formalin-fixed paraffin-embedded tissue sections (6 μm) were de-paraffinized with xylene for 30 min, rehydrated with graded alcohol, and then immersed in 0.1 M hydrochloric acid at 60°C for 5 min. Slides were then immersed in Schiff reagent for 30 min until the nuclei were stained, and then transferred directly to bisulfate water, followed by rinsing under running tap water. Following dehydration, the samples were treated with xylene, mounted in DPX and stored in shade. Nuclear morphometry was performed using a Nikon eclipse microscope (Nikon, Japan) connected with a Nikon CCD videocamera and an IBM Pentium 4/ PC with the appropriate Cell Measurement Software (Image Pro Plus v. 5.1, Media Cybernetics Inc, Silver Springs, MD, USA) as described previously by our group [9].

This analysis configuration permits operator-dependent selection and measurement of DNA content using a magnification of x200. Areas of the Feulgen-stained sections containing pathological lesions as defined by adjacent H&E stained slides were selected for DNA content analysis. A total of 200–300 nuclei with clear boundaries appearing to have no loss of membrane integrity were identified for analysis from each tissue sample, overlapping nuclei were excluded. By the calculation of configuration the software discharges the majority of overlapping nuclei, internal reference cells are selected and additional non-diagnostic nuclei are discarded by supplementary obligatory visual review. Reference cells’ coefficient of variation is limited to 5%, automatically. Gray levels in the microscopic image were transformed into digitalized signals and evaluated, with the image analysis system allowing differentiation between gray level intervals. Cytometrical measurements were calculated automatically according to the algorithms described previously by measuring the nuclear integrated optical density (IOD), which represents the cytometrical equivalent of its DNA content [10].

The procedure was performed for all nuclei and the overall mean represented DNA content or DNA index (DI). The mean IOD of control cells (human lymphocytes) served as the diploid standard (2c) and reference for DI calculation for targeted cells. Subsequently DNA histograms were generated. A tumour was classified as diploid if the DI ranged from 0.9 to 1.1 and the relevant DNA histogram revealed only 1 peak at 2c and aneuploid if any from the previous 2 criteria was absent.

### *Η. Pylori* serology

Sera from all patients were analysed for HP with an enzyme-linked immunosorbent assay (ELISA) IgG serologic test for (Allergy Immunotechnologies Inc., Newport Beach, CA, USA) in accordance to the manufacturer’s instructions. HP antibody titers higher than 155 mU/L were considered positive and lower than 155 mU/L negative. The specificity and sensitivity of the serology test has been estimated to be 95% and 90% [11].

### Statistical analysis

The primary end point of the study was Overall Survival (OS). A prognostic model was established by searching all variables that were significantly related to OS at a level of P values ≤ 0.05 in the univariate analysis. Descriptive Statistics were used to calculate frequencies and percentages for all variables involved. The Kaplan-Meier method was used to estimate the effect of the different variables on OS. Survival rates among categories (existence of factors or not) were compared for statistical differences using the Log-rank analysis. Multivariate analysis was subsequently carried out using stepwise Cox proportional hazards modelling for OS; best model was constructed using automated methods. Hazard ratio (HR) values together with the 95% Confidence interval (CI) are presented for all variables studied. All categorical variables were compared using a baseline category as reference. All analyses were conducted at a 5% significant level using SPSS v12.0 statistical package.

## Results

### Patients

218 cases of gastric cancer patients that have undergone surgery followed by chemotherapy were included in this analysis. The median age was 61 (Mean ± Standard Deviation (SD): 59 ± 9.53) years. Patient characteristics are presented in Table 1.

### Survival analysis

**Figure 1:**
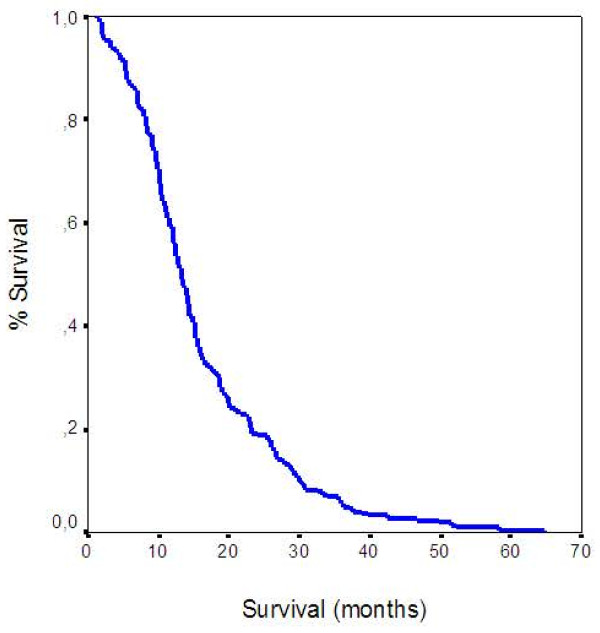
Overall Survival.

No patient was alive by the time of this analysis. OS was calculated for all patients in weeks. The 1-year Overall Survival was 51.38%. The median survival time for all patients was 13.25 months [95% Confidence Interval (CI) 12.00, 14.50] and the mean was 16.00 months [95% confidence Interval (CI) 14.50, 17.50] (Figure 1).

### Univariate analysis

At univariate analysis, using log rank tests seven of the parameters studied were found to adversely relate to survival (Table 2): PS (p < 0.001), presence of liver metastasis (p < 0.001), albumin ≤ 3.4 g/dL (P = 0.002), CRP >5 mg/dL (p < 0.001), CA19-9 >30U (p = 0.019), CA 72–4 >7 U/ml (p = 0.002) and DNA Index (p < 0.001). *H. Pylori* infection was not found to be significantly associated with OS (p = 0.35).

**Table 2:** Univariate Analysis of Survival

**UNIVARIATE ANALYSIS OF SURVIVAL**				
**Variable**		**MST**	**1-year survival %**	**P-value**
Age Category	≤ 60	16.50	53.00%	0.591
	>60	15.75	47.86%	
Gender	Male	15.75	50.34%	0.515
	Female	16.50	49.3%	
	60-70	11.75	30.30%	
Karnofsky PS	80	16.75	52.40%	<0.001
	90-100	19.25	63.20%	
	1	14.50	36.40%	0.092
Histological Grade	2	17.25	54.30%	
	3	14.25	44.87%	
Lymph node metastasis	No	13.50	28.89%	0.051
	Yes	16.75	56.65%	
Liver metastasis	No	19.25	63.39%	<0.001
	Yes	12.75	37.74%	
Lung metastasis	No	16.25	52.24%	0.185
	Yes	12.75	35.29%	
Ovarian metastasis	No	16.00	51.27%	0.579
	Yes	17.00	47.62%	
Bone metastasis	No	16.25	51.18%	0.282
	Yes	12.25	42.86%	
Peritoneal dissemination	No	16.75	54.90%	0.319
	Yes	15.00	43.70%	
Albumin	Normal	18.00	57.66%	0.002
	Low	12.75	38.30%	
CRP	Normal	18.00	56.06%	<0.001
	High	12.75	43.02%	
HP	No	15.50	48.59%	0.35
	Yes	17.25	52.63%	
CEA	≤ 5	15.00	45.54%	0.143
	> 5	17.00	52.83%	
CA 19-9	≤ 30	18.25	58.14%	0.019
	> 30	14.50	46.21%	
CA 72-4	≤ 7	21.75	65.00%	0.002
	> 7	14.75	47.46%	
	Low	22.50	73.86%	
DNA Index	Medium	12.75	35.20%	<0.001
	High	09.50	23.81%	

*MST* Median Survival Time, *PS* Performance Status, *CRP* C-Reactive Protein, *HP Helicobacter Pylori*, *CEA* Carcinoembryonic Antigen, *CA 19–9* Cancer Antigen 19–9, *CA 72–4* Cancer Antigen 72–4.

### Multivariate analysis

**Figure 2:**
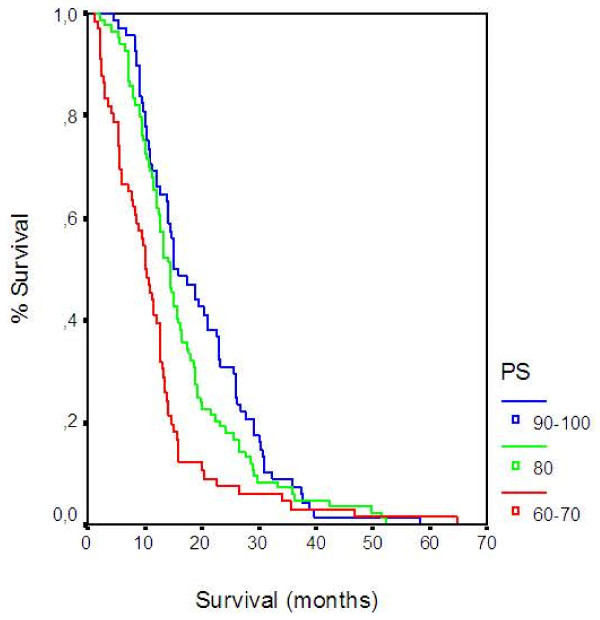
Overall Survival according to Performance Status.

**Figure 3:**
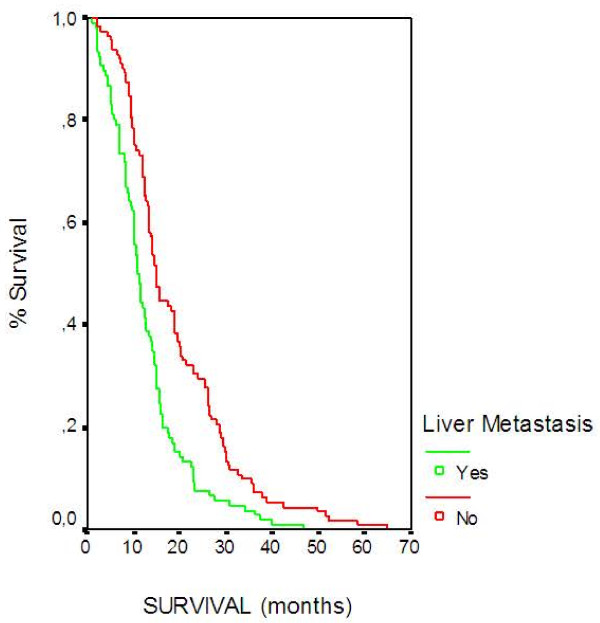
Overall Survival according to Liver metastasis.

**Figure 4:**
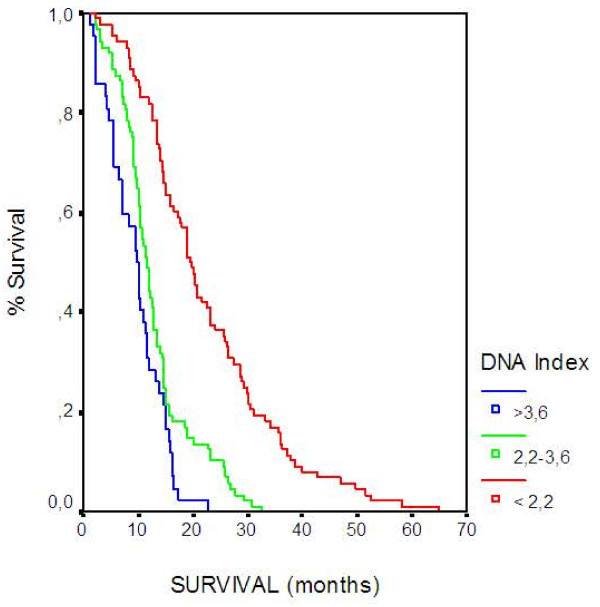
Overall Survival according to DNA Index.

Results of the multivariate analysis are presented in Table 3. Prognostic factors adversely affecting survival were PS, liver metastasis, and DNA Index. Based on the model, patients with PS 60–70 had 1.7 times higher possibility of death as compared to those with a PS 90–100 (HR 1.676; CI 1.171-2.398, p = 0.005), (Figure 2). The presence of liver metastasis was associated with 1.7 times higher possibility of death (HR 1.745; CI 1.318-2.310, p < 0.001), (Figure 3). Also, patients with DNA Index >3.6 have 4.2 times higher chance of death when compared to those with <2.2 (HR 4.207; CI 2.751-6.433 <0.001), (Figure 4) and patients with DNA Index 2.2-3.6 had a 3.05 higher probability of death when compared to those with <2.2 (HR 3.059; CI 2.185-4.283, p < 0.001), (Figure 4).

**Table 3:** Multivariate Analysis of Survival

	**P value**	**Hazard ratio (HR)**	**95% CI**	
			**Lower**	**Upper**
PS (60–70 *vs*. 90–100)	0.005	1.676	1.171	2.398
PS (80 *vs.* 90–100)	0.161	1.263	0.911	1.753
Liver Metastasis (Yes *vs.* No)	<0.001	1.745	1.318	2.310
DNA Index (2.2-3.6 *vs*. <2.2)	<0.001	3.059	2.185	4.282
DNA Index (>3.6 *vs.* <2.2)	<0.001	4.207	2.751	6.433

## Discussion

Following the publication of the Intergroup-0116 (INT-0116) and the MAGIC studies [12,13] which demonstrated a survival benefit from the combination of either postoperative chemoradiation or perioperative chemotherapy with surgery for resectable oesophagogastric cancer, a number of studies have suggested that the survival advantage conferred by this multimodality approach could be cautiously extended to patients with Stage IV disease. However, as this approach is potentially associated with high incidence of side effects it would be important to identify factors that predict survival to assist with patient selection and justify indication and feasibility.

Our retrospective analysis has evolved PS, liver metastasis and DNA content (DNA Index) as independent predictors of survival in our patient cohort which included patients with Stage IV gastric adenocarcinoma treated with palliative gastrectomy and systemic chemotherapy. HP infection was not identified as significant prognostic indicator by either univariate or multivariate analysis.

Although numerous studies report the detrimental effect of poor PS on survival in Stage IV gastric cancer, these mostly involve patients treated with palliative chemotherapy alone [14-16] like the one by Kim *et al.* which involved 304 consecutive patients with newly diagnosed metastatic or recurrent gastric cancer treated with one or more cycles of cisplatin-based chemotherapy [4]. We could only identify one study which assessed the prognostic significance of PS in a multimodality setting which involved intraperitoneal chemotherapy to treat intra-abdominal gross residual lesions after palliative gastrectomy with maximal cytoreduction. This particular study by Jeung *et al.* which involved 53 patients identified PS as the only significant defining factor for progression-free survival (P = 0.009) by multivariate analysis [17].

Liver metastasis is also a well-established prognostic indicator in patients with advanced gastric cancer treated with either palliative chemotherapy or surgery but not the combination. For example a pool analysis of 1080 chemotherapy naïve patients with locally advanced or metastatic oesophagogastric cancer by Chau *et al*. identified liver metastasis as an adverse prognostic factor by multivariate analysis [14]. Similarly, liver invasion has evolved as an independent survival indicator in a large prospective study involving 539 patients with advanced gastric cancer that had undergone surgical resection [18].

The prognostic value of DNA content in gastric cancer as measured by the DNA Index (DNA ploidy) is controversial as divergent DNA content analysis results have been reported by various studies. These are thought to reflect objective differences in the analytical techniques employed (image cytometry *vs.* flow cytometry) and intratumoural DNA ploidy heterogeneity. Image cytometry is considered superior to flow cytometry as it allows direct visualization and selection of tumour cells for inclusion in the DNA measurement. This qualitative feature appears to outbalance its lower throughput when compared with flow cytometry as was illustrated in a recent study by Belien *et al*. on the prognostic value of both image and flow cytometric analysis of DNA content in gastric cancer [19]. Their patient cohort consisted of 221 cases of gastric cancer analyzed for DNA content using the guidelines of the European consensus report on standardization of diagnostic DNA image cytometry and flow cytometry [20,21]. Although this study has demonstrated equal sensitivity for both methods in detecting DNA non-diploid gastric cancers, image cytometry DNA content analysis outperformed flow cytometry in predicting survival by multivariate analysis.

Other studies demonstrating the adverse prognostic significance of DNA analysis in advanced gastric cancer includes a study by Kimura *et al*. who analyzed the DNA content of 270 patients with advanced gastric cancer by flow cytometry to conclude that high DNA ploidy index was the third strongest prognostic factor for survival behind peritoneal dissemination and liver metastases (P < 0.01) [22]. A different study by Baba *et al.* including 93 patients with advanced gastric cancer showed that high DNA ploidy manifests with higher incidence of vessel invasion and lymph node metastasis invasion conferring a poor 5- year survival in elderly patients (P < 0.05) [23]. Similarly the flow cytometric analysis of gastric carcinomas performed by Danova *et al.* to evaluate ploidy patterns and the distribution of cells in the different cell cycle phases has demonstrated that DNA aneuploidy was a strong independent adverse prognostic factor for survival in patients with limited or advanced stage gastric cancer [24].

The relative consistency of reports on DNA Index analysis measurements in the few studies publishing results on advanced gastric cancer, irrespectively of the method applied, strengthens the validity of our data. This may be explained by the observation that aneuploidy appears to be more frequent in advanced gastric cancer and the majority of these aneuploid tumors are not DNA ploidy heterogeneous. That was depicted in a study by Osterheld *et al.* who performed DNA cytophotometry on multiple samples collected from 16 advanced gastric carcinomas and found 15 DNA-aneuploid tumours (94%) and one diploid tumour; multiple DNA-stemlines were found in 4 cases (26%). Furthermore, analysis of proliferative activity performed on the same samples revealed higher proliferation rate in DNA aneuploid homogeneous tumours than in aneuploid heterogeneous tumours and heterogeneous tumours did not overexpress p53. The authors suggested that the higher proliferative activity in homogeneous-aneuploid carcinomas and their more frequent overexpression of p53 support the hypothesis that in gastric cancer tumour progression implies the development of a dominant and more aggressive (higher proliferative activity, p53 overexpression) aneuploid cell clone [25]. This is particularly important as it has been shown that it is the DNA index of the subpopulation that is most widely distributed within gastric tumour is significantly associated with lymph node metastases (P < 0.001) and histologic grade (P < 0.001) [26].

Based on our analysis, the high DNA content (DNA Index) along with the poor PS and the presence of liver metastasis have evolved as independent predictors of survival in patients with Stage IV gastric adenocarcinoma treated with palliative gastrectomy and systemic chemotherapy. Since all these three variables can be assessed in preoperative biopsies, they may serve as a prognostic tool in order to preoperatively select patients eligible for palliative gastrectomy; however, prospective studies are needed to confirm this hypothesis.

There is considerable evidence to support causality between HP and sporadic noncardia gastric cancer [22]. For example, an individual-subject meta-analysis of 12 prospective serological studies including 1228 gastric cancer cases, in whom HP status was assessed by anti HP IgG antibodies with ELISA, reported that the relative risk of non cardia gastric cancer associated with HP infection is 5.9 [27]. It is generally accepted that HP infection leads to gastric cancer by inducing sequential alterations of the gastric mucosa, including chronic inflammation, atrophy and intestinal metaplasia, the latter considered as precancerous lesions. The extension of the preneoplastic lesions in the gastric mucosa although increases the risk for cancer development, creates an unfavorable environment for HP colonization and may account for the under-detection of HP infection in blood collected after the diagnosis of gastric cancer in case controlled studies [28]. In addition, it has been shown that conventional ELISAs used to assess serological presence of HP IgG antibodies in most epidemiological studies are likely to produce false-negative results for gastric cancer patients, as compared to population controls, which may further underestimate the risk [29,30]. In view of the above considerations, results from studies investigating the association of HP infection and prognosis in gastric cancer should be viewed with caution.

Meimarakis et al. used bacterial culture, histological analysis and serology (HP IgA and IgG ELISA) to assess HP status in 166 patients with gastric cancer and reported HP infection to be an independent prognostic factor for relapse free survival (HR 2.16, 95% CI 1.33-3.49) and OS (HR 2.00, 95%CI 1.22-3.57). Yet their patient cohort was different from ours as all their patients underwent curative resection (i.e. R0) followed by adjuvant treatment in 12 cases only (10 patients received intraoperative radiotherapy and 2 had postoperative chemotherapy with Cisplatin 5-FU and folinic acid); HP positivity was 75.3% [31]. Equivalent results were reported by Marrelli *et al* in a cohort of 297 patients with similar characteristics [32]. Furthermore, HP negative status (as assessed by histology) was reported to be the most significant independent prognostic factor of poor OS (HR 3.45, 95% CI: 2.43-4.89, p < 0.0001) in a different study which included only patients with locally advanced gastric cancer who underwent adjuvant chemotherapy after curative resection (≥D2 dissection) [33]. An attempt to explain the association between HP negativity and adverse survival implies a likely contribution of HP infection in augmenting anti-tumour immunity, especially in early stage gastric cancer [31], but this needs further validation.

However, in our study which included patients with advanced gastric cancer no association between HP infection status and survival was observed. HP seroprevalance was observed in only 34.9% of our patients as compared to approximately 65% expected for this age group (55–64 years) in Greece [34]. This may be related to the inferior methodology (ELISA) applied to assess HP positivity or it may simply depict mucosal changes and progressive pH alkalization in advanced gastric cancer that create a less favorable environment for HP colonization, as we mentioned above and has also been suggested by others [35]. Moreover, our findings are in agreement with a recent report by Qiu *et al* who used real-time PCR for HP detection in 157 gastric cancer patients and found no significant association between HP infection and OS or relapse-free survival in patients who underwent curative surgery [36].

The limitations of our study evolve around its retrospective nature and the objectivity of the methodologies used to assess key indicators such as HP status and DNA Index. Despite these limitations it has clinical relevance as we have validated a number of factors that could potentially be used to assess the likelihood of clinical benefit of a multimodal therapeutic approach with the combination of palliative gastrectomy and postoperative chemotherapy in gastric cancer patients with stage IV disease.

## Conclusion

In the present study a number of factors which could be used to predict survival in gastric cancer patients with Stage IV disease treated with palliative gastrectomy and postoperative chemotherapy have been identified. Poor pre-treatment PS, the presence of liver metastasis and high DNA Index were shown to be associated with adverse prognosis; the effect of HP infection status on survival has yet to be defined.

## Competing interests

The authors declare that they have no competing interests.

## Authors’ contributions

NT, SS, ESP and NK conceived of the study, participated in its design and coordination and helped to draft the manuscript. CK, JG, IK and EP participated in collection and interpretation of clinical data. GA participated in laboratory work, collection and interpretation of laboratory data. IDX and JS were involved in the design of the study, the collection and interpretation of clinical and laboratory data and drafted the manuscript. All authors read and approved the final manuscript.

## Pre-publication history

The pre-publication history for this paper can be accessed here:

http://www.biomedcentral.com/1471-2407/12/264/prepub
